# Computing systems, telehealth, and personal data: what is up?

**DOI:** 10.6061/clinics/2020/e2240

**Published:** 2020-11-02

**Authors:** Maria Inês Meurer

**Affiliations:** Universidade Federal de Santa Catarina, Centro de Ciencias da Saude, Florianopolis, SC, BR

Dear Editor,

I have read with great interest the comment published by the team coordinated by Prof. Paulo Sergio da Silva Santos (1), where the authors emphasized the importance of dentists (in particular a specialist in Oral Medicine) and Teledentistry strategies in the COVID-19 pandemic. I have been following the work of this nationally recognized team, known for their experience and excellence in the hospital environment, offering qualified dental care for patients with special medical needs. Personally, I have deep respect for their contribution to the multi-professional interaction in healthcare.

Considering the pandemic scenario, the authors highlighted, in their publication, the convenience of telehealth strategies in the exchange of health information. They also suggested the use of non-dedicated instant messaging and video calling applications to enable remote communication.

As an oral medicine specialist and a member of the team that has developed and recently launched a tele(oral) medicine service linked to the Brazilian Unified Public Health System (*Sistema Único de Saúde* *-* SUS) in the state of Santa Catarina (2), I would like to share some concerns about the use of non-dedicated platforms/softwares/applications for the exchange of health data.

Health data are considered sensitive data under the Brazilian General Data Protection Law (known as *Lei Geral de Proteção de Dados* - LGPD) (3). Sensitive data is a subcategory of personal data that represents a higher risk for the individual due to the discriminatory or harmful potential of its content (4). Similar attention is also given to sensitive data in the European Union (General Data Protection Regulation - GPDR) (5) and in the United States of America (Health Insurance Portability and Accountability Act - HIPAA) (6).

A number of requirements need to be met for the safe transit of sensitive health data through computer networks, which include the guarantee of confidentiality, authenticity, integrity, availability and non-retroactivity (timestamping) (7,8). Some additional processes are also involved depending on the situation, such as those related to digital signature and certification (7). Non-dedicated platforms/softwares/applications, despite their popularity and the eventual support of data encryption, are not the ideal choices for sharing sensitive data as they do not meet most of these requirements (9,10).

Information and communication technologies are increasingly impacting our personal and professional routines. Adapting to the changes imposed by technology requires expanding our field of knowledge (and thinking) beyond the health field.

The Brazilian LGPD comes into effect in September 2020. Since then, the use of systems/softwares/applications that do not meet the security requirements for sharing, archiving and deleting health data can result in major problems for health professionals. Thus, it is essential that we increase our understanding about these issues and change our practices to provide health care safely and ethically, through the virtual environment.

I appreciate the opportunity to respectfully and constructively share my point of view. May we all remain safe and healthy.
